# The complete genome of *Vibrio* sp. 16 unveils two circular chromosomes and a distinctive 46-kb plasmid

**DOI:** 10.1128/mra.01222-23

**Published:** 2024-02-28

**Authors:** Xiaofen Wu, Anca M. Segall, Carmela Giglione, Thierry Meinnel

**Affiliations:** 1 Université Paris-Saclay, CEA, CNRS, Institute for Integrative Biology of the Cell (I2BC), Gif sur Yvette, France; 2 Department of Biology, San Diego State University, San Diego, California, USA; 3 Viral Information Institute, San Diego State University, San Diego, California, USA; Portland State University, Oregon, USA

**Keywords:** *Vibrio*, bacteriophage evolution, genome analysis, plasmids, toxin/antitoxin systems

## Abstract

The entire 4.6-Mb genome of *Vibrio* sp. 16, encoding 4,270 genes, best matches with *Vibrio rotiferianus*. A 46-kb plasmid (pVDT1), alongside two circular chromosomes, showcases *parAB*/*repB* partition genes and three toxin/antitoxin systems potentially linked to phage infection.

## ANNOUNCEMENT

*Vibrio* sp. 16, also known as *Vibrio parahaemolyticus* strain 16 (1), is an unclassified *Vibrio* species (NCBI-Taxonomy #391586). It was isolated from surface waters in Tampa Bay, USA (latitude 27.96; longitude −82.46) and cultivated after streak isolation on marine agar. Serving as a host for numerous vibriophages, it contributes to modeling phage and bacterial evolution (1, 2). These phages play a crucial role in investigating the functions of peptide deformylase phage genes (3–6). Partial sequencing data are available (https://gold.jgi.doe.gov/analysis_project?id=Ga0031464).

*Vibrio* sp. 16 was provided by John Paul (University of South Florida). VDT1, a streptomycin-resistant isolate, was cultured overnight at 30°C with shaking (170 rpm) in liquid broth comprising 1% tryptone, 0.5% yeast extract, 3% NaCl, and 100 µg/mL streptomycin. DNA was extracted using the GenElute Bacterial Genomic DNA Kit (Sigma). Long-read sequencing libraries were prepared using the Native Barcoding kit SQK NBD114.24 (Oxford Nanopore Technologies) and sequenced on a Flo-Min114 Flow Cell (R10.4.1) on a GridION instrument (version GXB02022-23.07.5). Data analysis involved base calling and demultiplexing with Dorado 7.0.9 (https://github.com/nanoporetech/dorado), and long-read hierarchical assembly utilized Canu 2.1.1 (7). Genome display, circularization, format conversion, and curation were performed with SnapGene 7.0.3. Raw reads numbered 797,022 (4,511,949,794 bases) with N_50_ value of 8,256 bp. Genome annotation utilized PGAP 6.6 (8). Completeness used CheckM 1.2.2 (9). Default parameters were used for all software.

**Fig 1:**
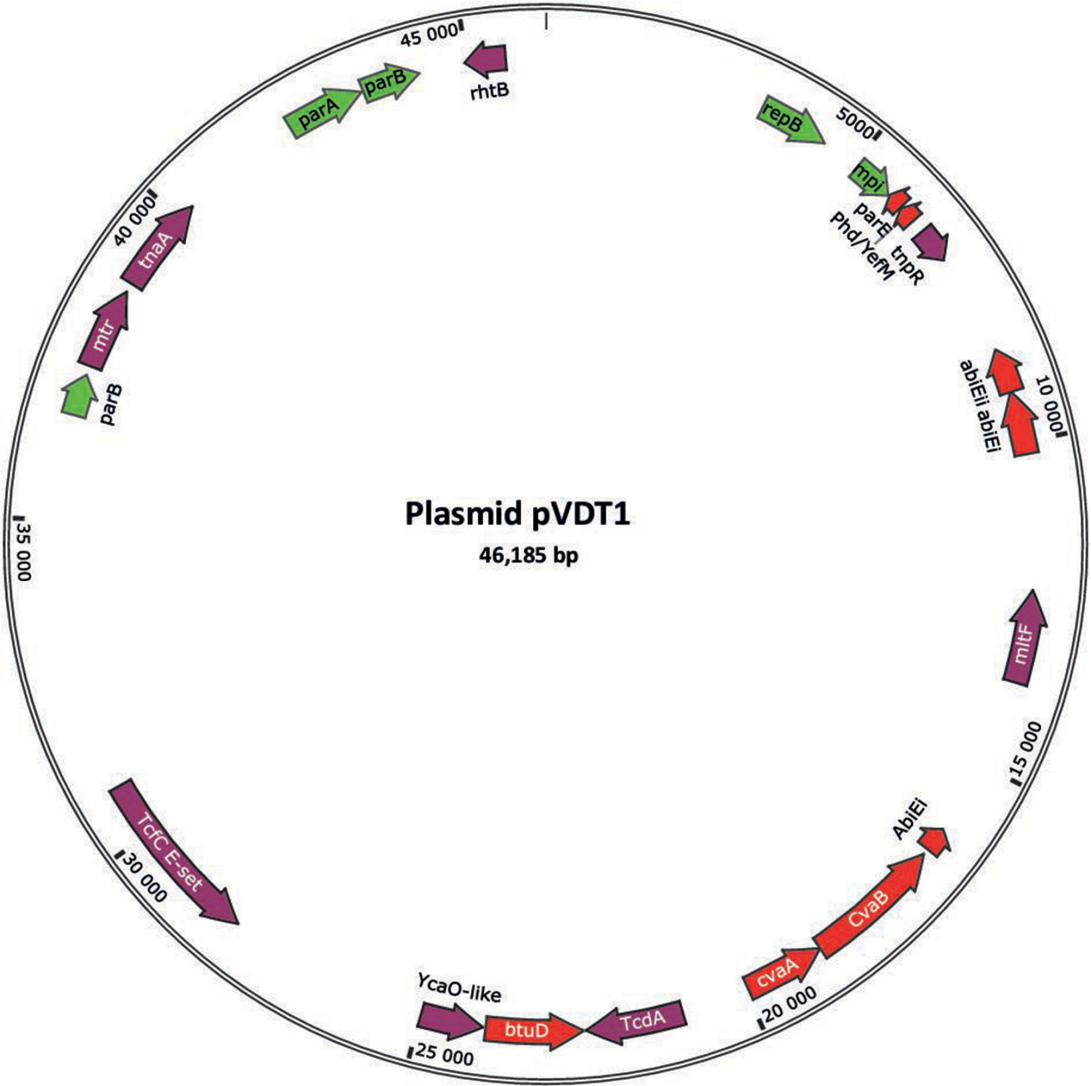
Simplified gene map of pVDT1. The circular map emphasizes 21 of the 53 coding sequences with homologies to known genes. Genes associated with toxin/antitoxin systems are marked in red, while those related to replication are highlighted in green. Twenty-four percent of the sequence exhibited 85.3% identity with regions near the replication origin, including *parAB* and *repB*, of an unnamed plasmid from *Vibrio campbellii* strain LJC014 (83.4 kb; GenBank ID CP050469.1).

Four contigs were generated. Circularization of chromosomes 1 and 2 was proposed by the Canu pipeline, suggesting repeat sequences on both ends (7). Trimming 22,073 and 23,485 bp, respectively, resulted in circular chromosomes of 3,080,496 bp and 1,531,398 bp. Circularization of contig 3 (89,403 bp) was indicated by BLAST alignment of the sequence against itself and dot plot analysis, revealing duplication of 43,218 bp. Validation involved comparing the overlap at the circularization end with partial genomic data (Table 1). The unique matching contig (scf_1108854221939; 47,116 bp) comprised a 931-bp overlap on each end. Trimming yielded the final 46,185-bp circular plasmid DNA, named pVDT1 (Fig. 1). The fourth contig, representing a linear 35-kb duplication of chromosome 2, was discarded. The complete genome size totaled 4,658,079 bp with a GC content of 46.3% (pVDT1 45%). The mean read depth for genome coverage was 945.

**TABLE 1:** *Vibrio* sp. 16 full genome annotation and comparison with partial sequencing data

Data	Complete genome	Partial genome^*a*^
Contigs	3	178^*b*^
Bases (bp)	4,658,079	4,488,436^*b*^
CheckM completeness (%)	99.65	93.25
Total gene number	4,270^*b*^	4,186
CoDing Sequences	4,092^*c*^	3,963
Total RNA genes	157	106
ncRNA genes^*d*^	1	1
rRNA genes^*d*^	34	27
tRNA genes^*d*^	119	75
Other RNA genes	3	3
Pseudogenes	21	117

^*a*^ Partial annotation data predicted with PGAP 6.6 are available at https://www.ncbi.nlm.nih.gov/datasets/gene/GCF_000158115.1/.

^*b*^ Only contig scf_1108854221952 (1,129 bp) matched neither *Vibrio* sp. 16 full genome nor any *Vibrio* full-length sequence. It likely corresponds to a bacterial DNA contamination as the sequence is 100% identical over the last 1,048 bp to *Escherichia coli* plasmids (e.g., CP083266.1), while bp 21–37 correspond to the M13 forward primer. In addition, the contig encodes the broad-spectrum beta-lactamase TEM-116 under control of the AmpR promoter, while *Vibrio* sp. 16 is sensitive to beta-lactams such as ampicillin.

^*c*^ VDT1 streptomycin resistance (0.1 g/L) may stem from the presence of Arg87 in the unique *rpsL* gene. Significantly, substituting the typically conserved Lys87 in enterobacterial *rpsL* genes with Arg has been associated with conferring streptomycin resistance (10).

^*d*^ ncRNA, rRNA and tRNA refer to non-coding, ribosomal and transfer RNA, respectively.

Average nucleotide identity analysis (11) revealed 99.99% identity of the partial sequence with the complete genome (see details in Table 1) and *Vibrio rotiferianus* as best match type-strain (85.19%). *V. rotiferianus* is a member of the Harveyi clade, which includes *V. parahaemolyticus* (12, 13). Only one peptide deformylase gene on chromosome 1 (*def1*) was observed, unlike other Vibrionaceae that display a *def2* gene on chromosome 2 (4, 10, 14). Several pVDT1 genes displayed strong sequence identity with *Vibrio* toxin/antitoxin systems genes (15), such as colicin V secretion CvaA/CvaB (99%/66%), type IV system AbiEi/AbiEii (87%/73%), or type II system YefM/ParE (98%/96%) family (Fig. 1). Toxin/antitoxin genes are implicated in bacterial virulence by inducing death of infected cells (16, 17) or in phage toxicity mediation (14). Thus, plasmid pVDT1 may play a role in controlling phage infectivity in *Vibrio* sp. 16.

## Data Availability

This Whole Genome Shotgun project has been deposited in the European Nucleotide Archive (ENA) at EMBL-EBI under accession number PRJEB69607. Raw data are available at https://www.ebi.ac.uk/ena/browser/view/ERR12308179. The annotated version described herein is the first RefSeq version currently available at https://www.ncbi.nlm.nih.gov/datasets/gene/GCF_963681195.1/.
